# Sustained Neutralizing Antibodies 6 Months Following Infection in 376 Japanese COVID-19 Survivors

**DOI:** 10.3389/fmicb.2021.661187

**Published:** 2021-05-07

**Authors:** Atsushi Goto, Hirofumi Go, Kei Miyakawa, Yutaro Yamaoka, Norihisa Ohtake, Sousuke Kubo, Sundararaj Stanleyraj Jeremiah, Takahiro Mihara, Kotaro Senuki, Tomoyuki Miyazaki, Satoshi Ikeda, Takashi Ogura, Hideaki Kato, Ikuro Matsuba, Naoko Sanno, Masaaki Miyakawa, Haruo Ozaki, Masakazu Kikuoka, Yasuo Ohashi, Akihide Ryo, Takeharu Yamanaka

**Affiliations:** ^1^Department of Health Data Science, Yokohama City University Graduate School of Data Science, Yokohama, Japan; ^2^Department of Biostatistics, Yokohama City University Graduate School of Medicine, Yokohama, Japan; ^3^Department of Microbiology, Yokohama City University Graduate School of Medicine, Yokohama, Japan; ^4^Life Science Laboratory, Technology and Development Division, Kanto Chemical Co, Inc., Kanagawa, Japan; ^5^Bioscience Division, Research and Development Department, Tosoh Corporation, Tokyo Research Center, Kanagawa, Japan; ^6^Advanced Medical Research Center, Yokohama City University, Yokohama, Japan; ^7^Department of Pulmonology, Yokohama City University Graduate School of Medicine, Yokohama, Japan; ^8^YCU Center for Novel and Exploratory Clinical Trials, Yokohama City University Hospital, Yokohama, Japan; ^9^Department of Physiology, Yokohama City University Graduate School of Medicine, Yokohama, Japan; ^10^Department of Respiratory Medicine, Kanagawa Cardiovascular and Respiratory Center, Yokohama, Japan; ^11^Infection Prevention and Control Department, Yokohama City University Hospital, Yokohama, Japan; ^12^Matsuba Medical Clinic, Kanagawa, Japan; ^13^Sanno Clinic Shinagawa, Tokyo, Japan; ^14^Japan Medical Association, Tokyo, Japan; ^15^Tokyo Medical Association, Tokyo, Japan; ^16^Kanagawa Prefecture Medical Association, Yokohama, Japan; ^17^Department of Integrated Science and Engineering for Sustainable Society, Chuo University, Tokyo, Japan

**Keywords:** SARS-CoV-2, COVID-19, immunity, neutralizing antibody, quantitative serological test

## Abstract

**Objective:** There is scarce evidence regarding the long-term persistence of neutralizing antibodies among coronavirus disease 2019 (COVID-19) survivors. This study determined neutralizing antibody titers (NT_50_) and antibodies against spike protein (SP) or nucleocapsid protein (NP) antigens approximately 6 months after the diagnosis of COVID-19.

**Methods:** COVID-19 survivors in Japan were recruited. Serum samples and data related to patients’ characteristics and COVID-19 history were collected. NT_50_ and titers of antibodies against NP and SP antigens were measured at 20–32 weeks after the first positive severe acute respiratory syndrome coronavirus 2 (SARS-CoV-2) test results. Factors associated with NT_50_ were identified using the multivariable linear regression and the correlations among NT_50_ and titers of immunoglobulin G (IgG) and total immunoglobulins (Igs) against NP and SP were assessed by Spearman’s correlation.

**Results:** Among 376 participants (median [range] days after testing positive for SARS-CoV-2, 180 (147–224); median [range] years of age, 50 (20–78); 188 [50%] male), most tested positive for NT_50_ (*n* = 367, 98%), SP-IgG (*n* = 344, 91%), SP-total Ig (*n* = 369, 98%), NP-IgG (*n* = 314, 84%), and NP-total Ig (*n* = 365, 97%). Regression analysis indicated that higher BMI, fever, and the requirement of mechanical ventilation or extracorporeal membrane oxygenation were significantly associated with higher NT_50_. Anti-SP antibodies correlated moderately with NT_50_ (Spearman’s correlation: 0.63 for SP IgG; 0.57 for SP-total Ig), while the correlation was weak for anti-NP antibodies (0.37 for NP IgG; 0.32 for NP-total Ig).

**Conclusions:** Most COVID-19 survivors had sustained neutralizing antibodies and tested positive for SP-total Ig and NP-total Ig approximately 6 months after infection.

## Introduction

The pandemic of coronavirus disease 2019 (COVID-19) is a serious threat to global health (The Johns Hopkins Coronavirus Resource Center, 2021). It started in Japan with the outbreak in the Diamond Princess Cruise Ship and continued to spread nationwide (Kato et al., 2020). There is hope that durable immunity, namely the so-called immunity passport, would develop after an infection, which is also expected to be achieved with vaccination (Phelan, 2020). Humoral immune responses, especially the production of neutralizing antibodies, play key roles in defending against severe acute respiratory syndrome coronavirus 2 (SARS-COV-2) infection (Stephens and McElrath, 2020). However, there is looming uncertainty regarding the duration and protective efficacy of the humoral immune response following primary infections. Various studies have reported a decline in neutralizing antibodies titers (Crawford et al., 2020; Gaebler et al., 2020; Long et al., 2020; Ripperger et al., 2020; Seow et al., 2020; Wajnberg et al., 2020; Wheatley et al., 2020) and also those of the antibodies against spike protein (SP) or nucleocapsid protein (NP) (Crawford et al., 2020; Gaebler et al., 2020; Ibarrondo et al., 2020; Kutsuna et al., 2020; Long et al., 2020; Patel et al., 2020; Ripperger et al., 2020; Seow et al., 2020; Wajnberg et al., 2020; Wheatley et al., 2020). However, these studies were limited by small sample sizes (Ibarrondo et al., 2020; Kutsuna et al., 2020) and most of the samples in these studies were collected within 2 months after infection (Long et al., 2020; Patel et al., 2020). The decline in antibody titers shown by these studies during the relatively early phase of recovery reflects a reduction in the number of short-lived plasmablasts, which is a natural process of immune response and should not be a concern (Stephens and McElrath, 2020). Subsequent to this decline, long-lived plasma cells in the bone marrow play active roles in maintaining circulating neutralizing antibodies (Stephens and McElrath, 2020). Considering the lacunae of the existing studies, there is a definitive need to determine the extent of duration the neutralizing antibodies persists following natural infection.

A large-scale study in Iceland reported that 1,107 of 1,215 (91.1%) COVID-19 survivors had positive pan-Ig assay results for SP and NP antigens 120 days after diagnosis (Gudbjartsson et al., 2020). However, the study lacked information regarding the NT_50_ and could not directly determine neutralizing activity persistence. A recent study with 36 samples of COVID-19 survivors at Mount Sinai Health System reported that robust neutralizing antibodies against SARS-CoV-2 infection persisted until 148 days post symptom onset (Wajnberg et al., 2020). However, those samples were selected according to the distribution of available enzyme-linked immunosorbent assay (ELISA) titers; they could therefore not reflect the entire study population. Thus, to the best of our knowledge, no definitive conclusion has been reached regarding the long-term sustainability of neutralizing antibodies after SARS-CoV-2 infection till date. Furthermore, clinical characteristics associated with neutralizing titers in the recovery stage of COVID-19 patients are yet to be understood.

Hence, in this cross-sectional study, we aimed to examine the humoral immune responses, including neutralizing antibodies, at 6 months after SARS-CoV-2 infection among 376 individuals. We studied the NT_50_ and the titers of antibodies against SP or NP antigens 6 months after the first positive results for SARS-CoV-2, to examine factors associated with NT_50_, and to investigate the correlation of the NT_50_ with the other antibody titers.

## Materials and Methods

### Study Design and Participants

Coronavirus disease 2019 survivors were recruited from the public through mass media advertisement via the news (television), social media, leaflets, and a website^1^ with the support of three Prefectural (Kanagawa, Tokyo, and Osaka, Japan) Medical Associations. Interested participants responded via a phone call. Eligible participants were aged ≥20 years at study entry time; residing in Japan; and had a positive result in either reverse transcription polymerase chain reaction (RT-PCR), loop-mediated isothermal amplification (LAMP), or antigen tests of pharyngeal swab, nasopharyngeal swab, or saliva for SARS-CoV-2. We excluded participants with fever of ≥37°C, cough, shortness of breath, sore throat, abnormalities of taste or smell, close contact with COVID-19 patients, or a history of foreign travel within 2 weeks of the scheduled blood collection date. A complete list of the inclusion and exclusion criteria is available at the University Hospital Medical Information Network-Clinical Trials Registry (UMIN-CTR), where this study was registered (number UMIN000041227; UMIN Clinical Trials Registry, 2021). The primary endpoints of this study were NT_50_ and titers of antibodies against NP and SP antigens at 20–32 weeks (visit 1) and 46–58 weeks (visit 2) after the first positive SARS-CoV-2 test results. Recruitment was limited to participants who could enroll at 20–32 weeks after the first positive SARS-CoV-2 test results. In this preliminary report, participants who enrolled and provided their serum samples from September 2nd to October 26th 2020 at visit one were included. This study is still ongoing and results including data from visit two will be reported later when the data become available. For all participants, physicians at cooperating outpatient clinics confirmed the diagnosis of COVID-19 based on information (e.g., medical records, medical certificate, a notice of work restriction, hospital admission admonishment, or SARS-CoV-2 test results of RT-PCR, LAMP, or antigen tests) provided by their hospitals, clinics, or public health centers. All participants provided written informed consent and the study was approved by the Institutional Review Board of Yokohama City University.

### Measurements

Data on participants’ characteristics (height and weight), lifestyle factors (smoking history), underlying conditions (cardiovascular diseases, cerebrovascular diseases, hypertension, diabetes, autoimmune diseases, active malignancy), date of the first positive test for SARS-CoV-2, date of onset of symptoms, symptoms (fever, cough, dyspnea, smell or taste disturbance, diarrhea), oxygen support requirements (any oxygen-supports, invasive mechanical ventilation, or extracorporeal membrane oxygenation [ECMO]), treatments (favipiravir, ciclesonide, steroids, and remdesivir), hospitalization, and dates of admission and discharge were collected during history taking by physicians at the clinics using case report forms. Body mass index (BMI) was calculated using self-reported height and weight: BMI = weight (kg)/height (m^2^).

For the neutralizing assay using pseudovirus, pseudotype lentivirus was produced by transient transfection of HEK293 cells with pNL4-3.Luc.R-E- and pSARS2-Spike-FLAG at a ratio of 1:1. Culture supernatants containing lentiviruses were collected 48 h after transfection and filtered through a 0.45-μm Millex-HV filter (Merck). VeroE6/TMPRSS2 cells seeded in 96-well plates were washed and inoculated with 100 μl of medium containing lentivirus stocks (20 μl) and five-fold serially diluted serum (1:50 to 1:31250 dilution). At 48 h after inoculation, the cells were washed and added to 40 μl of Bright-Glo Substrate (Promega). Luciferase activity was measured using the GloMax Discover System (Promega). The highest dilution of serum that resulted in a 50% reduction in luciferase luminescence compared with the non-serum control was set as the neutralizing titer (NT_50_). All samples were assayed in at least duplicate. We calculated NT_50_ using the curve fitting tool (Image J, version 1.52, NIH) with the bottom set to be 0 and the top set to be 1, as previously reported (Crawford et al., 2020). When serum had no observable neutralizing activity to interpolate NT_50_, it assigned a NT_50_ of 25. In a validation study, we confirmed that NT_50_ using the SARS-CoV-2 pseudovirus correlated well with NT_50_ using the authentic SARS-CoV-2 (Spearman’s correlation coefficients = 0.80; Supplementary Figure 1). The neutralizing assay using the authentic SARS-CoV-2 has been described in detail previously (Miyakawa et al., 2021). According to previous literature (Luchsinger et al., 2020; Seow et al., 2020), participants with NT_50_ of 51 or more were considered to have positive neutralizing activity. Participants were classified into five groups according to NT_50_ (undetectable: 0–50, low: 51–200, medium: 201–500, high: 501–2000, potent: 2000^+^), as previously reported (Seow et al., 2020).

Spike protein and NP antigens are widely used for detecting virus-specific antibodies. SP, a viral surface antigen, mediates the entry of SARS-CoV-2 into the host cells via RBD, while the NP antigens are non-surface antigens. IgG and total Ig (IgG, IgM, IgA, IgE, etc.) titers were measured using the advanced chemiluminescence enzyme immunoassay system AIA-CL1200 (Tosoh, Japan) against the ΔN-NP antigen (NP IgG and NP-total Ig) and RBD antigen (SP IgG and SP-total Ig) (Kubo et al., 2021). The truncated N-terminal NP (ΔN-NP), which is highly specific for detecting SARS-CoV-2, was used to detect antibodies to avoid cross-reactivity of NP (Yamaoka et al., 2020). The cut-off index of 1.0 was determined with the Youden’s J index to maximize the overall prediction performance. These four antibody measurements demonstrated sensitivity and specificity of 100% at ≥13 days after the onset of symptoms (Kubo et al., 2021). In addition, total Ig titers against NP antigens were measured using the Roche Elecsys Anti-SARS-CoV-2 electrochemiluminescence immunoassay (ECLIA) (Roche Diagnostics, 2021). The cut-off index of ≥1 was determined following the manufacturer’s instructions.

### Statistical Analysis

Characteristics of our study population are presented as medians for continuous variables and proportions for categorical variables according to NT_50_ groups. For NT_50_, nine participants had undetectable titers and were assigned the value of 25 for statistical analyses. For NP IgG, NP-total Ig, and SP IgG, seven participants had values below the limit of detection (LOD) and were assigned the value of 0.05, while five participants who had values below LOD for SP-total Ig were assigned 0.05. For Roche NP-total Ig, six participants had undetectable titers and were assigned 0.05 for statistical analyses. To compare the distribution of NT_50_ between groups for oxygen support need, box plots and nonparametric methods (Wilcoxon signed rank sum or Kruskal–Wallis) were used to compute *p*-values. Visual inspections of histograms revealed possible violations of the assumption of normality for antibody titers. Thus, all antibody titers were log (base 10)-transformed to improve normality assumptions. Thus, Spearman’s correlation was calculated to examine correlations among titers of neutralizing antibodies, NP IgG, NP-total Ig, Roche NP-total Ig, SP IgG, and SP-total Ig.

Simple and multiple linear regression analyses were performed to examine associations of participant characteristics with log_10_(NT_50_). Covariates included age, sex, days from the first positive SARS-Cov-2 test result, BMI, fever, cough, dyspnea, taste or smell disturbance, smoking status (never, current, or past smoking), oxygen support (none, required oxygen, or mechanical ventilation or ECMO), use of steroids or favipiravir, and history of diabetes. These covariates were chosen because of their potential association with NT_50_ based on previous literature (Wu F. et al., 2020) and clinical knowledge. Standard regression diagnostics (Fox and Weisberg, 2011) did not identify violation of regression assumptions. The thresholds for the statistical significance were set at a two-sided *p*-value < 0.05. All statistical analyses were performed using R, version 4.0.3.

## Results

Between September 2nd and October 26th, 2020, 376 participants with confirmed SARS-CoV-2 infection participated voluntarily in this study. The first date of positive SARS-Cov-2 test result ranged from February 4th to May 26th, 2020 (Supplementary Figure 2). The median (range) age of the participants was 50 (20–78) years, 188 (50%) participants were men, and the median (range) days from the first positive SARS-Cov-2 test result was 180 (147–224) days (Table 1). Among 376 participants, 71 (19%) underwent oxygen support and 25 (6.6%) underwent mechanical ventilation or ECMO during the clinical course. Those who underwent any oxygen support, mechanical ventilation, or ECMO were more likely to be male and older than those who did not. Fever was the most common symptom among 84% of overall participants, 79% had no oxygen support, 96% of participants who underwent oxygen therapy but did not undergo mechanical ventilation or ECMO, and 100% of those who received mechanical ventilation or ECMO had fever. The second common symptom, taste or smell disturbance, was found among 52% of all participants was relatively common among participants who did not undergo oxygen therapy (60%). Cough, the third common symptom, was found among 49% of all participants. Participants that received oxygen or ventilatory support tended to have diabetes.

**TABLE 1:** Characteristics of participants overall and with stratification by requirement of oxygen support (*N* = 376).

**Characteristic**	**Overall, *N* = 376**	**None, *N* = 280**	**Required oxygen^1^**, ***N* = 71**	**Mechanical ventilation or ECMO,** ***N* = 25**
Days from the first positive test	180 (147, 224)	180 (147, 224)	179 (148, 221)	179 (153, 220)
Age (years)	50 (20, 78)	47 (20, 78)	58 (26, 76)	59 (42, 72)
Men	188 (50%)	119 (42%)	48 (68%)	21 (84%)
BMI	23.2 (15.8, 42.4)	22.7 (15.8, 36.4)	24.0 (17.7, 42.4)	25.6 (17.0, 31.0)
Fever	314 (84%)	221 (79%)	68 (96%)	25 (100%)
Cough	184 (49%)	140 (50%)	36 (51%)	8 (32%)
Dyspnea	118 (31%)	71 (25%)	34 (48%)	13 (52%)
Taste or smell disturbance	196 (52%)	167 (60%)	24 (34%)	5 (20%)
Asymptomatic	15 (4.0%)	14 (5.0%)	1 (1.4%)	0 (0%)
Current smoking	34 (9.0%)	25 (8.9%)	5 (7.0%)	4 (16%)
Never smoking	235 (62%)	192 (69%)	31 (44%)	12 (48%)
Past smoking	107 (28%)	63 (22%)	35 (49%)	9 (36%)
Diabetes	30 (8.0%)	10 (3.6%)	15 (21%)	5 (20%)
Hospitalization	274 (73%)	178 (64%)	71 (100%)	25 (100%)
Favipiravir	85 (23%)	22 (7.9%)	42 (60%)	21 (84%)
Unknown	1	0	1	0
Remdesivir	5 (1.3%)	0 (0%)	3 (4.2%)	2 (8.0%)
Steroids	32 (8.5%)	7 (2.5%)	9 (13%)	16 (64%)
Unknown	1	0	1	0

*Statistics presented: median (range); *n* (%). BMI, body mass index; ECMO, extracorporeal membrane oxygenation; NT_50_, neutralizing titers. ^1^Participants who underwent oxygen therapy but did not undergo mechanical ventilation or ECMO.*

Overall, most participants tested positive for NT_50_ (367, 98%), SP-total Ig (369, 98%), SP-IgG (*n* = 344, 91%), NP-total Ig (*n* = 365, 97%), NP-IgG (*n* = 314, 84%), NP-total Ig (*n* = 365, 97%), and Roche NP-total Ig (*n* = 367, 98%) (Table 2). In addition, 85% (*n* = 317) had medium or higher NT_50_ (≥201). The positive rates of NT_50_ were 97% among participants without any oxygen support, 100% among those who received oxygen support or required mechanical ventilation or ECMO. Among asymptomatic participants who did not require any oxygen support (*N* = 14), 11 tested positive for NT_50_ (79%); among symptomatic participants who did not require any oxygen support (*N* = 266), 260 tested positive for NT_50_ (98%) (Supplementary Table 1).

**TABLE 2:** Distribution of NT_50_ and titers of antibodies against NP and SP antigens for among participants overall and with stratification by requirement of oxygen support (*N* = 376).

**Antibodies**	**Overall, *N* = 376**	**None, *N* = 280**	**Required oxygen^1^, *N* = 71**	**Mechanical ventilation or ECMO, *N* = 25**
**NT_50_ positive**	367 (98%)	271 (97%)	71 (100%)	25 (100%)
**NT_50_ groups**				
Undetectable	9 (2.4%)	9 (3.2%)	0 (0%)	0 (0%)
51–200	50 (13%)	45 (16%)	5 (7.0%)	0 (0%)
201–500	180 (48%)	139 (50%)	35 (49%)	6 (24%)
501–2000	132 (35%)	86 (31%)	30 (42%)	16 (64%)
2000+	5 (1.3%)	1 (0.4%)	1 (1.4%)	3 (12%)
**SP-IgG positive**	344 (91%)	248 (89%)	71 (100%)	25 (100%)
**SP-Total Ig positive**	369 (98%)	273 (98%)	71 (100%)	25 (100%)
**NP-IgG positive**	314 (84%)	222 (79%)	67 (94%)	25 (100%)
**NP-Total Ig positive**	365 (97%)	269 (96%)	71 (100%)	25 (100%)
**Roche NP-Total Ig positive**	367 (98%)	271 (97%)	71 (100%)	25 (100%)

*NT_50_, neutralizing titers. ^1^Participants who underwent oxygen therapy but did not undergo mechanical ventilation or ECMO.*

**FIGURE 1:**
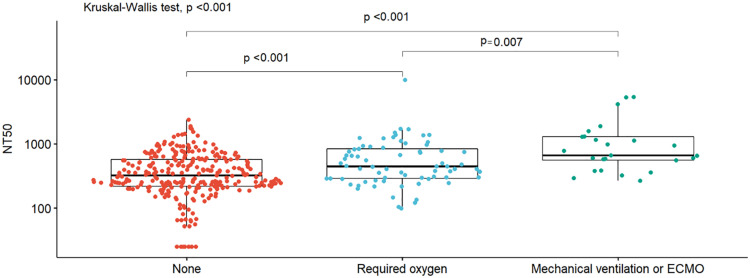
Box plots of NT_50_ titers by oxygen support. Box plots of neutralizing titers (NT_50_) by oxygen support requirements (none, required oxygen, invasive mechanical ventilation, or extracorporeal membrane oxygenation [ECMO]), with the density distribution of the data within each group, are shown. Differences in distributions between groups were assessed using nonparametric methods (Wilcoxon signed rank sum or Kruskal–Wallis).

Participants who received oxygen had significantly higher NT_50_ than those who did not; those that required mechanical ventilation or ECMO had significantly higher NT_50_ than those who received oxygen alone (Figure 1). We further stratified participants according to five groups according to NT_50_. Among those without oxygen support, those with higher NT_50_ tended to have symptoms such as fever and cough, higher BMI, and diabetes; and used favipiravir and steroids (Supplementary Table 2A). Among participants who received oxygen support but did not undergo mechanical ventilation or ECMO, those with higher NT_50_ tended to have higher BMI (Supplementary Table 2B). Among participants who required mechanical ventilation or ECMO, no factors seemed to be correlated with NT_50_ (Supplementary Table 2C).

**FIGURE 2:**
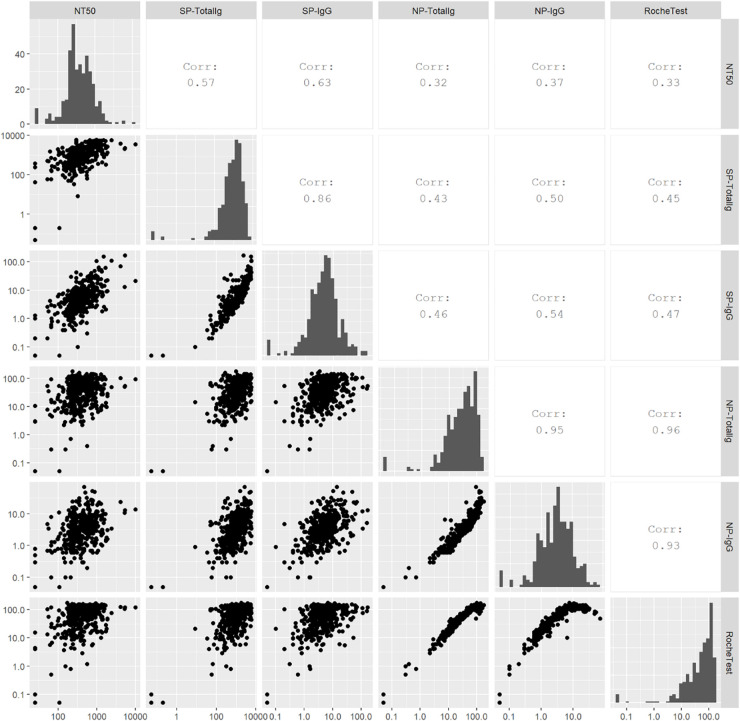
Scatter plots, Spearman’s correlations, and histograms for antibody titers. Spearman’s correlation was calculated to examine correlations among neutralizing titers (NT_50_), SP-total Ig, SP IgG, NP-total Ig, NP IgG, and Roche NP-total Ig. Higher value represents stronger correlation.

In the multivariable-adjusted model, higher BMI, fever, and the requirement of mechanical ventilation or ECMO were significantly associated with higher titers (Table 3). The NT_50_ did not differ by days from the first positive results of the SARS-CoV-2 test (Supplementary Figure 3). Spearman’s correlation coefficients between NT_50_ and antibodies against SP or NP antigens were the strongest for NT_50_ vs SP IgG (*r* = 0.63), followed by NT_50_ vs SP-total Ig (*r* = 0.57), while antibodies against NP antigen showed weak correlations with NT_50_ (*r* = 0.37 for NT_50_ vs NP IgG; *r* = 0.32 for NT_50_ vs NP total Ig; *r* = 0.33 for NT_50_ vs Roche NP-total Ig) (Figure 2).

**TABLE 3:** Linear regression of log_10_(NT_50_) titers on participant characteristics (*N* = 375).

**Variables**	**Crude models^1^**			**Adjusted model^1^**		
	**Beta**	**95% CI**	***p*-value**	**Beta**	**95% CI**	***p*-value**
Age (in 10-year increments)	0.06	0.03to0.09	<0.001	0.03	0.00to0.06	0.087
Men	0.09	0.01to0.17	0.021	–0.03	−0.11to0.06	0.51
Days from the first positive test (in 30-day increments)	–0.01	−0.09to0.08	0.89	–0.01	−0.09to0.07	0.81
BMI (in 5-unit increments)	0.10	0.06to0.15	<0.001	0.08	0.02to0.13	0.004
Fever	0.17	0.07to0.28	<0.001	0.11	0.01to0.21	0.028
Cough	0.04	−0.04to0.11	0.35	0.03	−0.05to0.10	0.46
Dyspnea	0.08	0.00to0.16	0.066	0.02	−0.07to0.10	0.72
Taste or smell disturbance	–0.06	−0.13to0.02	0.15	0.03	−0.05to0.10	0.45
Smoking status						
Current smoking vs never smoking	–0.06	−0.20to0.07	0.37	–0.10	−0.23to0.03	0.13
Past smoking vs never smoking	0.04	−0.05to0.12	0.43	–0.04	−0.12to0.05	0.40
Oxygen support						
Oxygen vs none	0.19	0.10to0.28	<0.001	0.06	−0.06to0.19	0.30
Mechanical ventilation or ECMO vs. none	0.43	0.28to0.57	<0.001	0.24	0.05to0.44	0.016
Diabetes	0.25	0.11to0.39	<0.001	0.09	−0.05to0.24	0.20
Favipiravir	0.24	0.15to0.33	<0.001	0.09	−0.02to0.21	0.097
Steroids	0.29	0.15to0.42	<0.001	0.06	−0.09to0.22	0.43

*Simple and multiple linear regression analyses were performed to examine associations of participant characteristics with log_10_(NT_50_). One with missing on steroid use and favipiravir use was not used in the regression analyses. CI: confidence interval; ECMO: extracorporeal membrane oxygenation; log_10_: Log (base 10)-transformation; NT_50_: neutralizing titers. ^1^In the crude models, one of the variables listed was included as an independent variable in a simple linear regression model. In the adjusted model, all variables listed were included as independent variables in a multiple linear regression model.*

## Discussion

This cross-sectional study of 376 participants across Japan provides evidence that approximately 98% COVID-19 survivors had sustained NT_50_ (≥51) 6 months after infection. Furthermore, 97% of participants who did not undergo any oxygen support, 100% of those with oxygen support had positive NT_50_. Those who underwent mechanical ventilation or ECMO were more likely to have higher NT_50_. In addition, most participants (97 to 98%) tested positive for SP-total Ig, NP-total Ig, and Roche NP-total Ig. These findings provide insight into long-term humoral immune responses after COVID-19 infection, supporting the notion that neutralizing activity remains at detectable levels in most COVID-19 survivors.

With respect to the duration of persistence of neutralizing antibodies, the findings our study are in line with those of certain studies reported previously (Wajnberg et al., 2020; Wu J. et al., 2020). In a study comprising 36 COVID-19 survivors, Wajnberg et al. (2020) have shown that despite their early decline from day 30 until day 82 after infection, NT_50_ persisted until day 148 and concluded that robust neutralizing antibody responses to SARS-CoV-2 infection persist for months following infection. Seow et al. (2020) have also pointed out the early waning of neutralizing responses to SARS-CoV-2 infection. In this study from the UK, the NT_50_ were found to wane at day 40 or later following the onset of COVID-19 symptoms and had reduced titers at 94 days after symptom onset (Seow et al., 2020). This decline in neutralizing antibodies at a relatively early phase of infection has been consistently reported in many studies with varieties of populations (Crawford et al., 2020; Gaebler et al., 2020; Ibarrondo et al., 2020; Kutsuna et al., 2020; Long et al., 2020; Patel et al., 2020; Ripperger et al., 2020; Seow et al., 2020; Wajnberg et al., 2020; Wheatley et al., 2020) and is known to occur after the acute phase of infection (Stephens and McElrath, 2020). The crucial question is whether the neutralizing antibodies titers persist long term during the recovery phase, despite the early waning phase. The positivity rate of relatively long-term neutralizing antibodies during the convalescent phase has not been well-documented in large patient cohorts. Our findings provide evidence that 98% of survivors had sustained neutralizing antibody titers 6 months after infection.

Consistent with previous studies (Seow et al., 2020), participants who underwent mechanical ventilation or ECMO were more likely to have higher NT_50_ than those who did not. We also found that higher BMI and fever were significantly associated with higher NT_50_. Participants with more severe disease might have had higher viral loads, possibly inducing higher levels of viral antigen and more potent immune responses (Seow et al., 2020; Wu F. et al., 2020). Another possibility is that higher titers of antibodies could have induced more severe disease. Surprisingly, in our study, most participants who did not require oxygen support (97%) had neutralizing antibodies, suggesting that milder infections could also induce sustained immune responses. These findings might be relevant for vaccine development.

Spearman’s correlation between antibody titers revealed moderate correlations for SP IgG vs NT_50_ (*r* = 0.63) and SP-total Ig vs NT_50_ (*r* = 0.57). The imperfect correlation denotes that the titers of SP-IgG or SP-total Ig identify only the amount of antibodies, whereas NT_50_ evaluates the neutralizing activity of antibodies. Hence the presence of SP IgG does not always denote the presence of neutralizing antibodies. The strengths of the correlations in our study were weaker than those of the correlations reported in the Mount Sinai study (Wajnberg et al., 2020). A stronger correlation for titers of IgG against SP by ELISA (i.e., the Mount Sinai ELISA) vs NT_50_ (*r* = 0.79) at day 148 after infection was reported. Our assays for SP IgG and SP-total Ig were designed to detect antibodies against RBD antigen (Kubo et al., 2021); however, the Mount Sinai ELISA consisted of two direct serial assays against RBD and SP (Amanat et al., 2020). The differences in the assays may explain the discrepancy between the correlations. Our assays showed high sensitivity in the acute phase of the infection. The present study showed that the sensitivity (i.e., positive rates) for all the four assays was ≥84% at 6 months after the infection. In particular, SP-total Ig and NP-total Ig showed high sensitivity of 98 and 97%, respectively. Because highly sensitive and specific assays are needed to accurately estimate the frequency of previous SARS-CoV-2 infections, SP-total Ig and NP-total Ig assays are useful for seroprevalence studies.

Some limitations merit consideration. First, we could not investigate the longitudinal changes in antibody titers because of the cross-sectional design of this study. Thus, the antibody titers in our participants might decline over time. Our observation of the participants’ immune responses at a certain period strongly suggests that most exhibited potent immune responses. Second, participants recruited from the community via the media might have resulted in a healthy volunteer bias. However, the frequency (64.9%, 178/274) of participants who did not receive oxygen among hospitalized participants in our study was similar to that (61.6%, 1623/2636) reported in the COVID-19 registry in Japan (Matsunaga et al., 2020). Thus, we believe that the selection bias was not a serious concern. Third, clinical information was collected through history taking by physicians during visits to the clinics using case report forms. Because the data were not directly collected from hospitals where the participants were diagnosed or treated for COVID-19, recall bias could not be ruled out. However, because participants and clinical staffs were unaware of their antibody titers at the time of study entry, the possible bias was probably non-differential and the results of the regression analyses reported in Table 3 might have been biased toward the null. Finally, because of the inherent nature of cross-sectional study designs, we could not establish a temporal relationship between participant characteristics (e.g., oxygen support and use of steroids, etc.) and antibody titers.

In conclusion, this cross-sectional study of 376 participants revealed that most COVID-19 survivors had sustained neutralizing antibody titers 6 months after infection. Furthermore, almost all participants tested positive for SP-total Ig and NP-total Ig. These findings add to the evidence that COVID-19 survivors have humoral immune responses 6 months after infection. These data would help clinicians and public health professionals to guide their communities regarding the management and prevention of COVID-19.

## Data Availability Statement

The datasets presented in this article are not readily available because it is difficult to ensure de-identification of data. However, they can be available from the corresponding author on reasonable request. Requests to access the datasets should be directed to TY, yamanaka@yokohama-cu.ac.jp.

## Ethics Statement

The studies involving human participants were reviewed and approved by the Institutional Review Board of Yokohama City University. The patients/participants provided their written informed consent to participate in this study. Written informed consent was obtained from the individual(s) for the publication of any potentially identifiable images or data included in this article.

## Author Contributions

AG contributed to the study design, data collection, statistical analysis and interpretation of data, and the drafting and editing of the manuscript. HG, TaM, and KS contributed to the study design and data collection. KM, NO, YY, and SK performed the laboratory tests. AR and TY contributed to the study design, data collection, and supervised the analysis and preparation of the manuscript. All authors made critical revisions to the manuscript for important intellectual content and approved the final manuscript.

## Conflict of Interest

NO is an employee of Tosoh Corporation. YY is an employee of Kanto Chemical Co., Inc. The remaining authors declare that the research was conducted in the absence of any commercial or financial relationships that could be construed as a potential conflict of interest.

## Footnotes

1 https://covid19-kaifuku.jp/

## Supplementary Material

Supplementary Figure 1 Scatterplot of neutralizing titers (NT_50_) calculated from the neutralization analysis using the SARS-CoV-2 pseudovirus against NT_50_ calculated from the assay using the authentic SARS-CoV-2 in a validation study (Spearman’s correlation coefficients = 0.80).

Supplementary Figure 2 Number of participants by date of test for COVID-19 diagnosis. Histogram for number of participants by date of test for COVID-19 diagnosis is shown.

Supplementary Figure 3 Scatterplot of NT_50_ against days from the first positive test. Scatterplot of neutralizing titers against days from the first positive test for COVID-19 diagnosis is shown. NT_50_: neutralizing titers.
