# Supplementary material for: Sustained Neutralizing Antibodies 6 Months Following Infection in 376 Japanese COVID-19 Survivors
Source: Front Microbiol. 2021 May 7;12:661187. doi: 10.3389/fmicb.2021.661187 (PMC8137897; doi:10.3389/fmicb.2021.661187)

Supplementary Material

Supplementary Table 1 Distributions of NT_{50} and titers of antibodies against NP and SP antigens with stratification by the presence of any symptoms and requirement of oxygen support (N=376)

Antibodies	Asymptomatic, no oxygen	Symptomatic, no oxygen	Required oxygen, [1]	Mechanical ventilation or ECMO
	N = 14	N = 266	N = 71	N = 25
**NT_{50} positive**	11 (79%)	260 (98%)	71 (100%)	25 (100%)
**NT_{50} groups**				
Undetectable	3 (21%)	6 (2.3%)	0 (0%)	0 (0%)
51-200	3 (21%)	42 (16%)	5 (7.0%)	0 (0%)
201-500	5 (36%)	134 (50%)	35 (49%)	6 (24%)
501-2000	3 (21%)	83 (31%)	30 (42%)	16 (64%)
2000+	0 (0%)	1 (0.4%)	1 (1.4%)	3 (12%)
**SP-IgG positive**	8 (57%)	240 (90%)	71 (100%)	25 (100%)
**SP-Total Ig positive**	11 (79%)	262 (98%)	71 (100%)	25 (100%)
**NP-IgG positive**	7 (50%)	215 (81%)	67 (94%)	25 (100%)
**NP-Total Ig positive**	10 (71%)	259 (97%)	71 (100%)	25 (100%)
**Roche NP-Total Ig positive**	10 (71%)	261 (98%)	71 (100%)	25 (100%)

ECMO: extracorporeal membrane oxygenation; NT_{50}: neutralizing titers.

Supplementary Table 2A. Characteristics of participants according to categories of NT_{50} among those who did not require oxygen support (N=280)

Characteristic	Undetectable, N = 9	51-200, N = 45	201-500, N = 139	501-2000, N = 86	2000+, N = 1
Days from the first positive test	179 (163, 216)	178 (154, 213)	181 (148, 217)	180 (147, 224)	186 (186, 186)
Age (years)	36 (21, 73)	46 (22, 66)	45 (20, 78)	50 (21, 72)	42 (42, 42)
Men	3 (33%)	15 (33%)	58 (42%)	43 (50%)	0 (0%)
BMI	19.6 (17.6, 26.4)	21.5 (16.0, 29.4)	22.9 (15.8, 36.3)	23.5 (17.4, 36.4)	23.5 (23.5, 23.5)
Fever	4 (44%)	33 (73%)	112 (81%)	71 (83%)	1 (100%)
Cough	4 (44%)	18 (40%)	73 (53%)	44 (51%)	1 (100%)
Dyspnea	1 (11%)	11 (24%)	34 (24%)	24 (28%)	1 (100%)
Taste or smell disturbance	2 (22%)	34 (76%)	83 (60%)	47 (55%)	1 (100%)
Asymptomatic	3 (33%)	3 (6.7%)	5 (3.6%)	3 (3.5%)	0 (0%)
Current smoking	1 (11%)	5 (11%)	14 (10%)	5 (5.8%)	0 (0%)
Never smoking	7 (78%)	30 (67%)	99 (71%)	55 (64%)	1 (100%)
Past smoking	1 (11%)	10 (22%)	26 (19%)	26 (30%)	0 (0%)
Diabetes	0 (0%)	0 (0%)	3 (2.2%)	7 (8.1%)	0 (0%)
Hospitalization	8 (89%)	29 (64%)	87 (63%)	54 (63%)	0 (0%)
Favipiravir	0 (0%)	3 (6.7%)	10 (7.2%)	9 (10%)	0 (0%)
Remdesivir	0 (0%)	0 (0%)	0 (0%)	0 (0%)	0 (0%)
Steroids	0 (0%)	0 (0%)	2 (1.4%)	5 (5.8%)	0 (0%)

Statistics presented: median (range); n (%). BMI: body mass index; NT_{50}: neutralizing titers.

Supplementary Table 2B. Characteristics of participants according to categories of NT_{50} among those with oxygen support without mechanical ventilation or ECMO (N=71)

Characteristic	Undetectable, N = 0	51-200, N = 5	201-500, N = 35	501-2000, N = 30	2000+, N = 1
Days from the first positive test	NA	178 (158, 181)	180 (148, 221)	178 (153, 220)	178 (178, 178)
Age (years)	NA	54 (48, 60)	57 (26, 76)	58 (39, 76)	59 (59, 59)
Men	NA	4 (80%)	23 (66%)	20 (67%)	1 (100%)
BMI	NA	23.1 (19.4, 29.4)	23.4 (18.1, 29.4)	24.5 (17.7, 42.4)	29.4 (29.4, 29.4)
Fever	NA	5 (100%)	34 (97%)	28 (93%)	1 (100%)
Cough	NA	3 (60%)	18 (51%)	14 (47%)	1 (100%)
Dyspnea	NA	4 (80%)	13 (37%)	17 (57%)	0 (0%)
Taste or smell disturbance	NA	2 (40%)	12 (34%)	9 (30%)	1 (100%)
Asymptomatic	NA	0 (0%)	0 (0%)	1 (3.3%)	0 (0%)
Current smoking	NA	1 (20%)	3 (8.6%)	1 (3.3%)	0 (0%)
Never smoking	NA	0 (0%)	13 (37%)	17 (57%)	1 (100%)
Past smoking	NA	4 (80%)	19 (54%)	12 (40%)	0 (0%)
Diabetes	NA	0 (0%)	9 (26%)	5 (17%)	1 (100%)
Hospitalization	NA	5 (100%)	35 (100%)	30 (100%)	1 (100%)
Favipiravir	NA	3 (60%)	17 (50%)	21 (70%)	1 (100%)
Unknown	NA	0	1	0	0
Remdesivir	NA	1 (20%)	1 (2.9%)	1 (3.3%)	0 (0%)
Steroids	NA	1 (20%)	2 (5.9%)	6 (20%)	0 (0%)
Unknown	NA	0	1	0	0

Statistics presented: median (range); n (%). BMI: body mass index; NT_{50}: neutralizing titers; ECMO: extracorporeal membrane oxygenation.

Supplementary Table 2C. Characteristics of participants according to categories of NT_{50} among those with mechanical ventilation or ECMO (N=25)

Characteristic	Undetectable, N = 0	51-200, N = 0	201-500, N = 6	501-2000, N = 16	2000+, N = 3
Days from the first positive test	NA	NA	178 (153, 208)	182 (166, 220)	168 (164, 177)
Age (years)	NA	NA	60 (47, 69)	60 (42, 72)	59 (56, 68)
Men	NA	NA	6 (100%)	13 (81%)	2 (67%)
BMI	NA	NA	25.3 (22.6, 27.1)	27.2 (17.0, 31.0)	23.5 (21.7, 24.5)
Fever	NA	NA	6 (100%)	16 (100%)	3 (100%)
Cough	NA	NA	3 (50%)	4 (25%)	1 (33%)
Dyspnea	NA	NA	5 (83%)	8 (50%)	0 (0%)
Taste or smell disturbance	NA	NA	0 (0%)	5 (31%)	0 (0%)
Asymptomatic	NA	NA	0 (0%)	0 (0%)	0 (0%)
Current smoking	NA	NA	1 (17%)	3 (19%)	0 (0%)
Never smoking	NA	NA	2 (33%)	7 (44%)	3 (100%)
Past smoking	NA	NA	3 (50%)	6 (38%)	0 (0%)
Diabetes	NA	NA	1 (17%)	3 (19%)	1 (33%)
Hospitalization	NA	NA	6 (100%)	16 (100%)	3 (100%)
Favipiravir	NA	NA	5 (83%)	13 (81%)	3 (100%)
Remdesivir	NA	NA	1 (17%)	1 (6.2%)	0 (0%)
Steroids	NA	NA	5 (83%)	9 (56%)	2 (67%)

Statistics presented: median (range); n (%). BMI: body mass index; NT_{50}: neutralizing titers; ECMO: extracorporeal membrane oxygenation; NA: not applicable.

Supplementary Figure 1

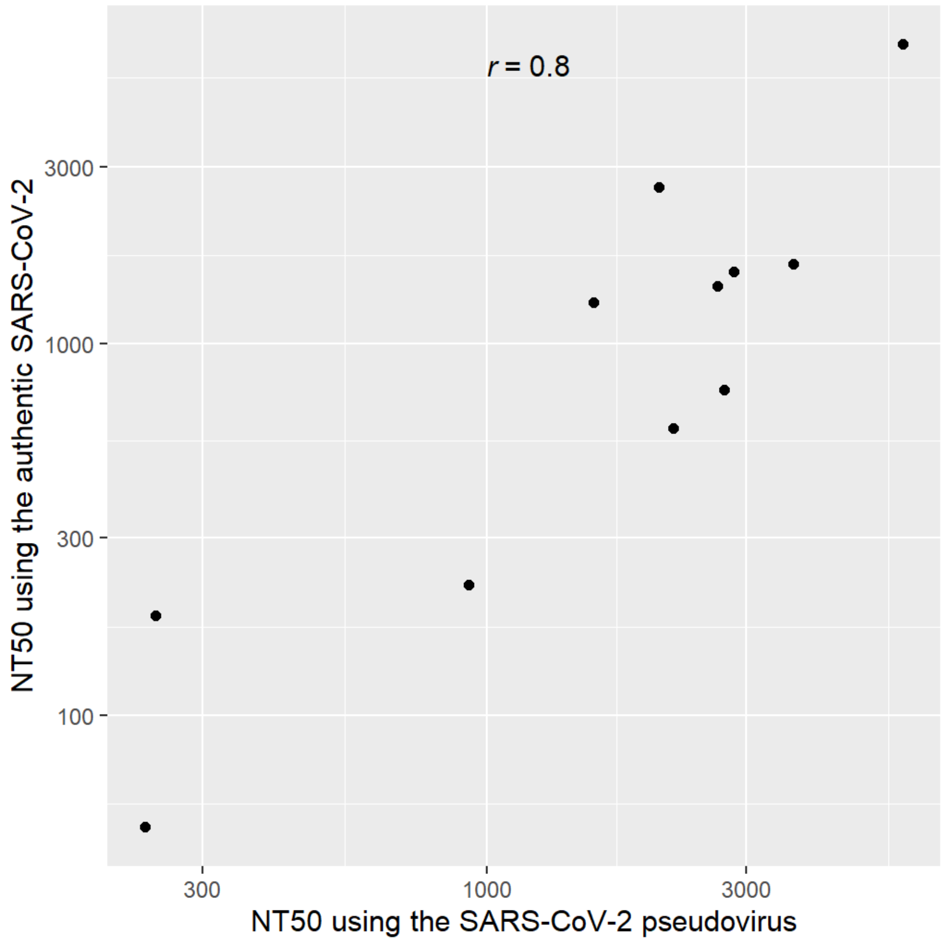

Supplementary Figure 2.

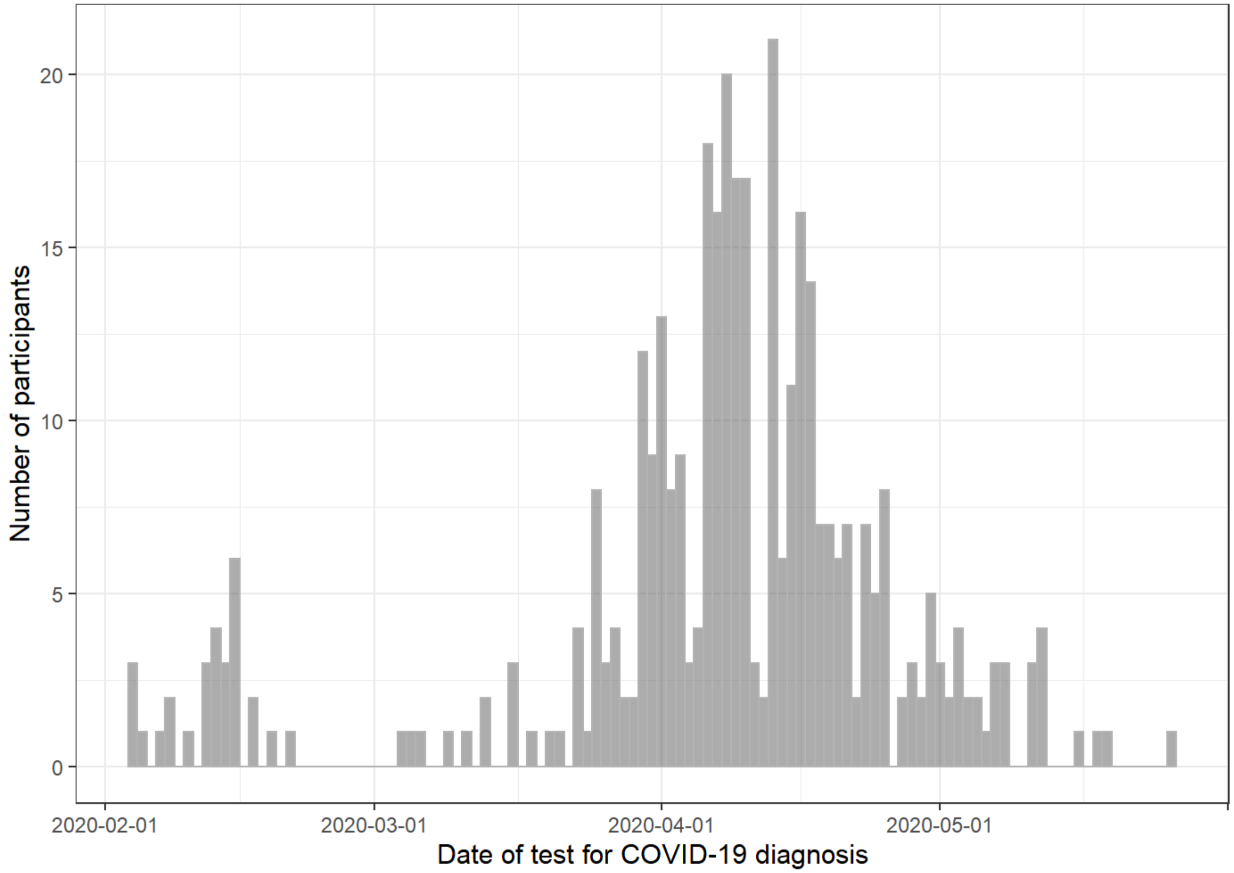

Supplementary Figure 3.

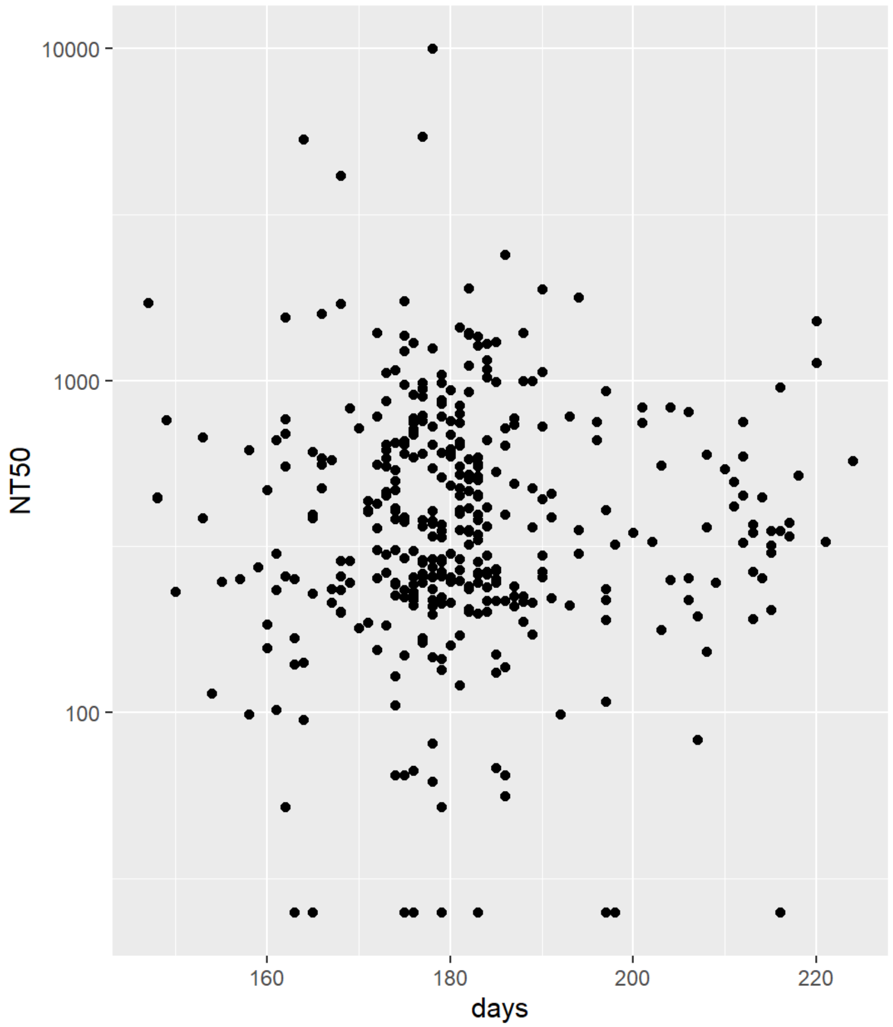

Supplement: Supplementary Figure 1 — Scatterplot of neutralizing titers (NT50) calculated from the neutralization analysis using the SARS-CoV-2 pseudovirus against NT50 calculated from the assay using the authentic SARS-CoV-2 in a validation study (Spearman’s correlation coefficients = 0.80). [file Data_Sheet_1.pdf]
